# Evolving new ways to secure a mate

**DOI:** 10.7554/eLife.78246

**Published:** 2022-04-05

**Authors:** Xiaodan Lin, Dong Ren

**Affiliations:** 1 Key Laboratory of Green Prevention and Control of Tropical Plant Diseases and Pests, Ministry of Education, College of Plant Protection, Hainan University Haikou China; 2 College of Life Sciences and Academy for Multidisciplinary Studies, Capital Normal University Beijing China

**Keywords:** mating strategies, coercive copulation, amber, Mecoptera, sexual conflict, Mesozoic, Other

## Abstract

Fossils shed light on mating strategies in scorpionflies.

**Related research article** Soszyńska-Maj A, Krzemińska E, Pérez-de la Fuente R, Wang JS, Szpila K, Skibińska K, Kopeć K, Krzemiński W. 2022. Evolution of sexual conflict in scorpionflies. *eLife* **11**:e70508. doi: 10.7554/eLife.70508.

During the Mesozoic period, when dinosaurs roamed the Earth, an order of flying insects called the Mecoptera were abundant in many parts of the world. These insects are between two and thirty-five millimeters long, and are commonly referred to as ‘scorpionflies’ because males have a large penis that sits above their body and resembles the stinger of a scorpion.

Currently, the fossil record of Mecoptera shows evidence for 39 families, but only nine of these have survived until the present (Lin et al., 2019; Wang and Hua, 2018). These families include the Panorpidae (which has 480 species, making it the largest extant family), the Panorpodidae (also known as short-faced scorpionflies), the Bittacidae (also known as hangingflies), the Choristidae and the Eomeropidae (which contains just one species). The origin and evolution of Mecoptera are still not fully understood, which is why these insects have long fascinated entomologists, naturalists and evolutionary biologists.

Researchers speculate that the key to understanding the evolution of Mecoptera is to understand their unique feeding and mating behaviors (Gao et al., 2021). For instance, scorpionflies have diverse feeding habits that allow them to eat a variety of foods, ranging from vegetation to other dead or live insects (Palmer, 2010). As a result, some families, such as the Panorpidae and Bittacidae, have chewing mouthparts for solid food, while others have sucking mouthparts for feeding on nectar or other plant liquids (Ren et al., 2009; Lin et al., 2019; Zhao et al., 2020). While having a particular diet allows scorpionflies to adapt better to their surroundings, it also makes them more susceptible to environmental changes. This may explain why some species became extinct during the Cretaceous period when the Earth’s geology and climate underwent a significant shift, 145–66 million years ago.

The mating behaviors of scorpionflies are also diverse and flexible, with males employing two main strategies: gift giving and coercive copulation. In some extant families – including the Panorpidae, Bittacidae and Choristidae – the males provide gifts to the females before or during mating in order to ensure completion of copulation (Tong and Hua, 2019). This gift can be saliva or, in species with less-developed salivary glands, prey – such as a small insect – that has been killed by the male (Zhong and Hua, 2013). In the other main strategy, coercive copulation, males rely on modifications to their genitalia and other parts of their body to hold females in place by force during mating.

One reason why male scorpionflies have developed a range of mating strategies is that they suffer more from sexual conflict than females. This is when a mating strategy that favors one sex puts the other at a disadvantage: in scorpionflies, for example, females make more reproductive sacrifices during mating and are therefore more selective when choosing a partner compared to males, who prefer to have as many partners as possible. As a result, males face a large amount of competitive pressure from other males, and also from females, so they employ various coercive copulation or gift giving strategies to relieve this sexual conflict.

The success of coercive copulation relies on the specialization of the genitalia, the organ that evolves most rapidly in males (Rowe and Arnqvist, 2012). However, non-genital structures that contact the female during mating, such as the notal and postnotal organs along the abdomen, also have an important role. These organs allow the male to hold different body parts of the female during mating to increase the copulation time. They are particularly well-developed in species that use coercive copulation, but are relatively small or absent in those that employ gift giving as a mating strategy (Tong and Hua, 2019).

Coercive copulation and gift giving have both had prominent roles in the evolution of mating strategies for Mecoptera. Now, in eLife, Agnieszka Soszyńska-Maj (University of Łódź), Ewa Krzemińska (Polish Academy of Sciences) and co-workers report the discovery of new genitalia and non-genital structures, some of which have not been seen in either extant or extinct mecopterans before (Soszyńska-Maj et al., 2022). The team – who are based at various institutes in Poland, the United Kingdom and China – discovered three new species of Mecoptera in amber deposits from Myanmar, the Baltics and Spain, which reflect the diverse mating behaviors of these insects (Figure 1).

**Figure 1.**
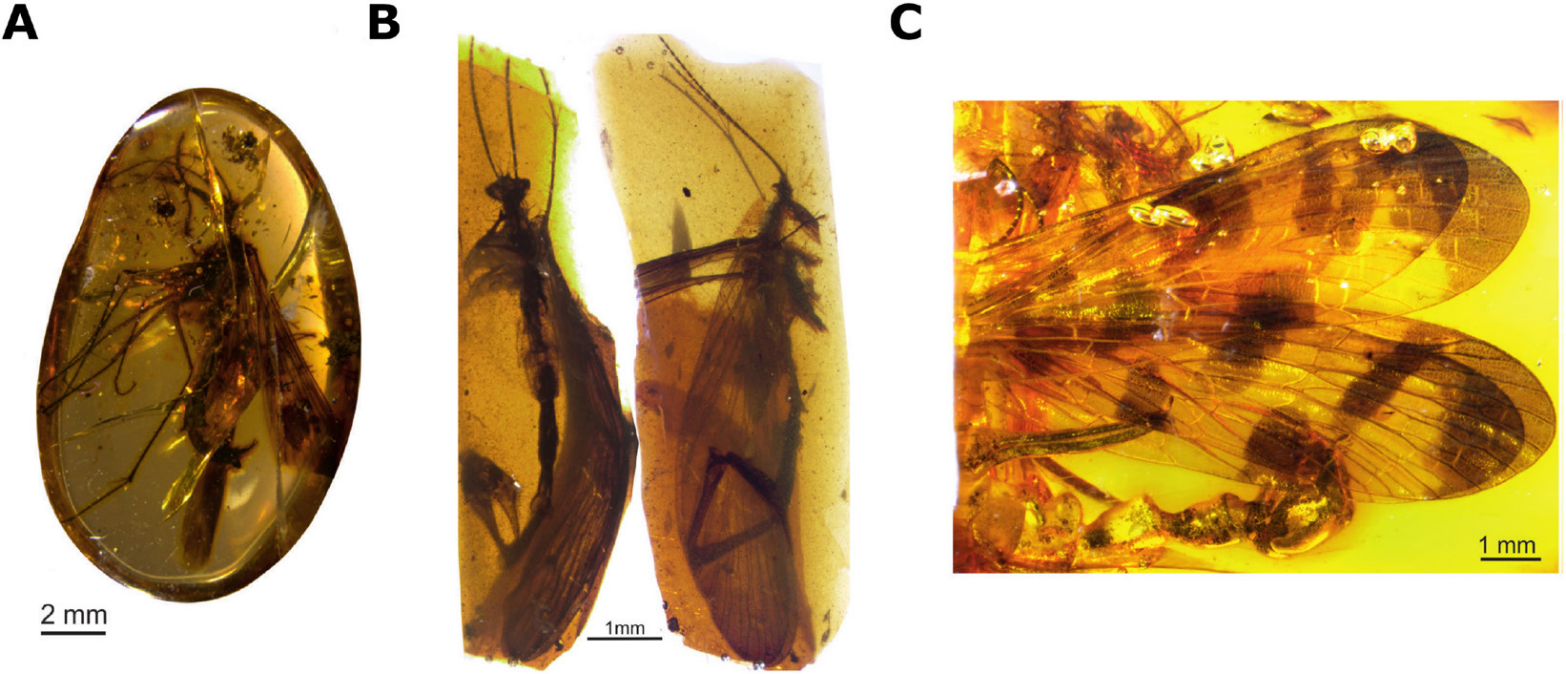
New species of extinct scorpionflies. Soszyńska-Maj et al. found three new species of extinct scorpionflies in amber deposits from Myanmar, Spain and the Baltics: *Burmorthophlebia multiprocessa* (**A**)*, Cantabra soplao* (**B**), and *Baltipanorpa oppressiva* (**C**). Studying anatomical features on the abdomens of these species revealed whether the males relied more on coercive copulation or gift giving as a mating strategy.

Image credit: Adapted from Figures 1A, 2A and 3A in Soszyńska-Maj et al., 2022.

Each specimen had different abdominal features that provide clues as to the mating strategy they may have used. For example, the scorpionfly discovered in Spain, named *Cantabra soplao* (Cantabridae), had a small postnotal organ and no other abdominal processes, suggesting it relied more on gift giving than coercive copulation to secure a mate. In contrast, *Baltipanorpa oppressiva* (Panorpidae), which was found in the Baltics, had very long postnotal and notal organs that act as a sort of clamp which holds the female wings in place (Figure 2). At the end of the organs was a fork-like structure that had not been seen before which ‘clips’ the clamp together, making this one of the most effective restraining devices ever found on a member of the Panorpidae family. The third specimen, *Burmorthophlebia multiprocessa* (Orthophlebiidae), had a similar clamp; however, it was relatively small, suggesting this species likely used a combination of gift giving and coercive copulation.

**Figure 2.**
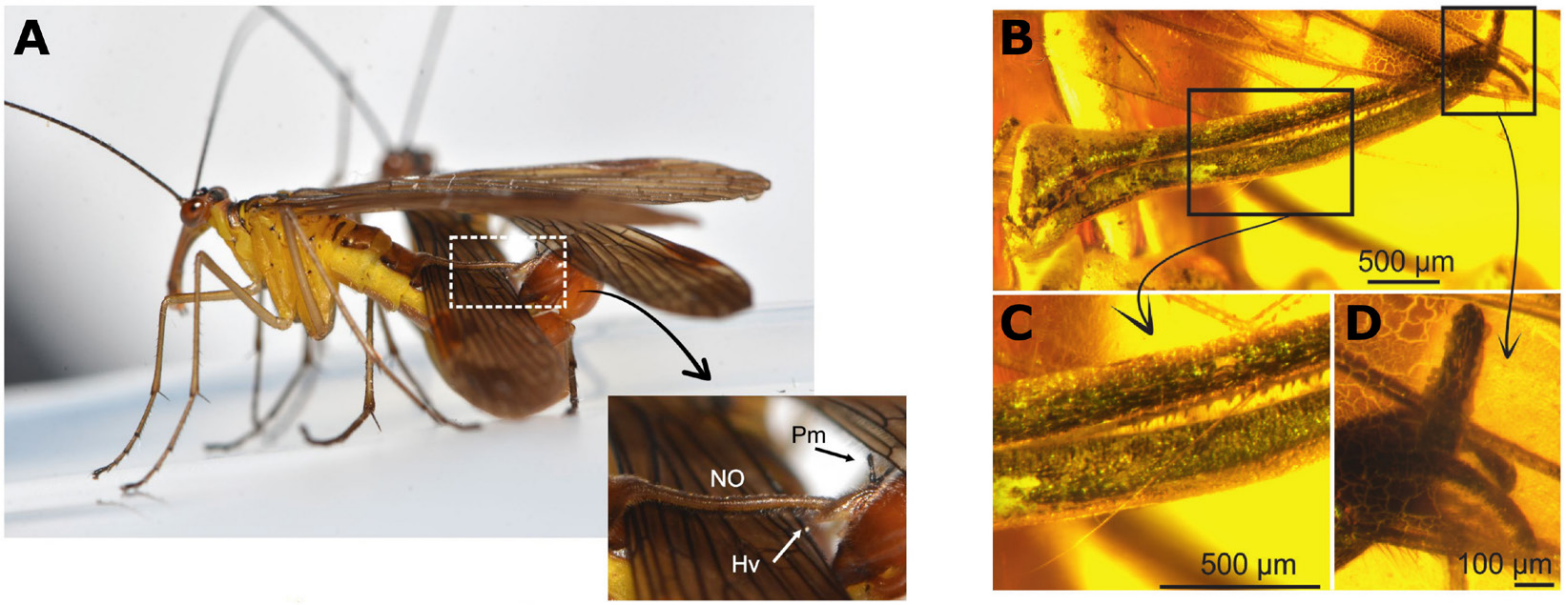
The unique clamp of *Baltipanorpa oppressiva*. (**A**) Some male scorpionflies have various anatomical features on their abdomens that help hold the female in place during mating to increase the duration of copulation. These features includes the notal and postnotal organs, long spike-like structures which form a sort of clamp around parts of the female’s body, like her wings (outlined by white-dashed box). At the end of the clamp is part of the male’s external genitalia called the paramere, and the terminal region (or hypovalve) of the postnotal organ which can be seen appearing from underneath the female’s wing. (**B**) *Baltipanorpa oppressiva* has a similar clamp-like structure. However, the fly was found to have features that had not been seen before in other species, such as teeth inside the clamp (**C**), and a clasp at the end which helps ‘clip’ the two organs together (**D**). Abbreviations: NO, notal organ; Hv, hypovalve; Pm, paramere.

Image credit: Adapted from Figure 6A and Figure 1C, D and E in Soszyńska-Maj et al., 2022.

The findings of Soszyńska-Maj et al., together with previous studies reporting the morphology of other mecopterans (Lin et al., 2019; Zhao et al., 2020), suggest that the mating behavior of scorpionflies may have evolved through two distinct trends. First, was the evolution of the gripping structures that males use for coercive copulation, which are not present in groups that appeared early on in the tree of life, such as Eomeropidae (Figure 3A). Members of these ‘early’ families probably did not partake in gift giving due to the high-energy cost and complexity of this approach: thus, in combination with a lack of powerful gripping structures, this caused the efficiency and duration of their mating to suffer as a result.

**Figure 3.**
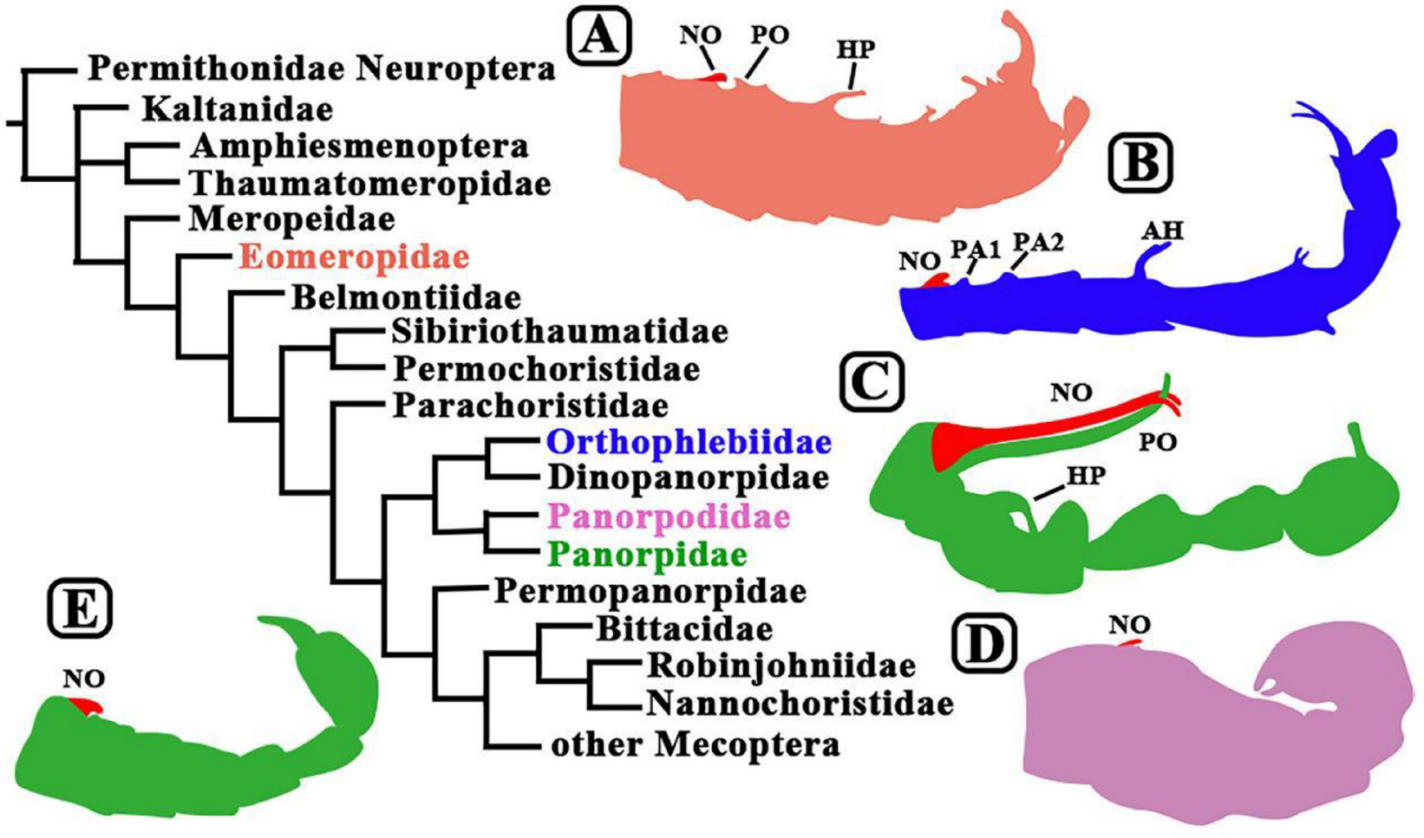
Evolution and mating behavior in Mecoptera. Scorpionflies and hangingflies belong to the order Mecoptera, which contains nine living families at present, but the fossil record shows that it has contained 39 families over time, some of which are shown in this phylogenetic tree (Lin et al., 2019; Zhao et al., 2020). The illustrations (which are not to scale) show the different ways in which the abdomen of male mecopterans have evolved. *Notiothauma reedi* (**A**) is a member of the Eomeropidae: like other early species, the male abdomen has not evolved the gripping structures that enable the males of some species to restrain females during mating (Mickoleit, 1971). *Burmorthophlebia multiprocessa* (**B**) and *Baltipanorpa oppressiva* (**C**) are members of the Orthophlebiidae and the Panorpidae respectively: both were recently discovered by Soszyńska-Maj et al., and in both species the male abdomen has evolved complex gripping structures that allow them to keep hold of females during coercive copulation. *Panorpodes kuandianensis* (**D**; Zhong et al., 2011; Wang and Hua, 2018) and *Panorpa jinhuaensis* (**E**; Wang et al., 2019) are members of the Panorpodidae and the Panorpidae respectively: the males of both species rely on gifts to attract mates, so have quite small gripping structures on their abdomens. It should be noted that the Panorpidae contains species that rely on coercive mating (**C**) and species that use gifts to attract mates (**E**). Abbreviations: NO, notal organ; PO, postnotal organ; AH, anal horn; PA1/PA2, postnotal area 1/postnotal area 2; HP, horn-like process.

Image credit: Xiaodan Lin and Dong Ren (CC BY 4.0).

As time went on, some species – such as *Burmorthophlebia multiprocessa* (Orthophlebiidae) and *Baltipanorpa oppressiva* (Panorpidae) *–* evolved reproductive and anatomical features that allowed them to adopt a more forceful mating strategy (Figure 3B and C). However, in some groups, these organs are degraded and not as complex, and the males instead have larger secretory organs so they can deliver better salivary gifts (Figure 3D and E). This represents the second trend: the rising popularity of gift giving, the strategy most commonly employed by extant species, including members of the Panorpidae and Panorpodidae families. These two trends may explain why the mating behavior of scorpionflies is so varied, particularly among species in the Panorpidae family (Zhong and Hua, 2013).

The new specimens and mating patterns described by Soszyńska-Maj et al. help us to better understand the evolutionary process of reproductive behaviors in Mecoptera. But there is still only a limited amount of material from which these conclusions can be drawn. As a result, many questions still remain. For instance, what are the evolutionary relationships between reproductive and non-reproductive structures in different families, or even among different genera and species in the same group?

It is likely that more specimens are yet to be discovered which, together with advancing research methods, will provide more clues as to how scorpionflies evolved their unique behaviors. This will not only be relevant for understanding the evolutionary biology of Mecoptera, but also how sexual conflict influenced the evolution of other orders of insects.
